# *Coriandrum sativum* and Its Utility in Psychiatric Disorders

**DOI:** 10.3390/molecules28145314

**Published:** 2023-07-10

**Authors:** Anislada Santibáñez, Enrique Jiménez-Ferrer, Paola Isabel Angulo-Bejarano, Ashutosh Sharma, Maribel Herrera-Ruiz

**Affiliations:** 1 Centro de Investigación Biomédica del Sur, Instituto Mexicano del Seguro Social, Argentina No. 1 Col Centro, Xochitepec 62790, Morelos, Mexico; anisszg@gmail.com (A.S.); enriqueferrer_mx@yahoo.com (E.J.-F.); 2 Plant Innovation Lab, Tecnologico de Monterrey, School of Engineering and Sciences, Centro de Bioingeniería, Av. Epigmenio González No. 500, San Pablo 76130, Queretaro, Mexico; pangulobe@tec.mx

**Keywords:** *Coriandrum sativum*, psychiatric disorders, anxiety, depression, epilepsy

## Abstract

The negative impact on worldwide social well-being by the increasing rate of psychiatric diseases has led to a continuous new drug search. Even though the current therapeutic options exert their activity on multiple neurological targets, these have various adverse effects, causing treatment abandonment. Recent research has shown that *Coriandrum sativum* offers a rich source of metabolites, mainly terpenes and flavonoids, as useful agents against central nervous system disorders, with remarkable in vitro and in vivo activities on models related to these pathologies. Furthermore, studies have revealed that some compounds exhibit a chemical interaction with γ-aminobutyric acid, 5-hydroxytryptamine, and N-methyl-D-aspartate receptors, which are key components in the pathophysiology associated with psychiatric and neurological diseases. The current clinical evaluations of standardized extracts of *C. sativum* are scarce; however, one or more of its compounds represents an area of opportunity to test the efficacy of the plant as an anxiolytic, antidepressant, antiepileptic, or sleep enhancer. For this, the aim of the review was based on the pharmacological activities offered by the compounds identified and isolated from coriander and the processes involved in achieving their effect. In addition, lines of technological research, like molecular docking and nanoparticles, are proposed for the future development of phytomedicines, based on the bioactive molecules of *C. sativum*, for the treatment of psychiatric and neurological disorders addressed in the present study.

## 1. Introduction

In 2019, the World Health Organization (WHO) pointed out a growing presence of mental disorders, increasing the burden that significantly impacts society’s health, affecting the economy of all countries [1]. This emphasizes human rights since many of these diseases are stigmatized, leading to greater suffering for patients and their families. The WHO details the different types of mental disorders characterized by the combination of thoughts, perceptions, emotions, behavioral changes, and abnormal social relationships. These disorders include depression, bipolar, anxiety, psychosis, dementia, and developmental disorders [1,2].

Although the strategies that this organization marks to stop the impact of this group of diseases are wide and varied, drug therapies that directly impact health are also sought, increasing the life quality of patients. That is why the chemical and pharmacological research of new treatments continues to be of paramount importance. Because medicinal plants are a source of secondary metabolites with multiple and varied biological activities that affect human health, they continue to be objects of study.

*Coriandrum sativum*, also known as coriander, is a species widely used throughout the world, mainly for its culinary usefulness, giving flavor to innumerable dishes in almost all cultures on the planet, which have included it since its endemic origin or because of its expansion. In addition, the much-appreciated “cilantro” has been characterized by a wide range of medicinal uses, among which stands out those that involve mental health, including psychiatric and neurological disorders that represent a cause of relevant health care for most of the societies around the globe [3,4].

In this review, we will mark some aspects of *C. sativum*. Even when many of its essential characteristics are widely known, we will focus on the biological actions of the plant that frame its ability to counteract or reduce mental health conditions. To date, there are no clinical studies on the activity of coriander on psychiatric or neurological conditions. Its clinical effectiveness is only mentioned in a randomized, triple-blind, placebo-controlled study, where patients with migraine received syrup from the fruit of coriander and sodium valproate. The authors of the study conclude that the intake of coriander causes a reduction in the duration, severity, and frequency of the condition [5].

It has been reported that extracts and essential oil of coriander leaves and seeds exert an anxiolytic, antidepressant, and sedative effect in pre-clinical models with zebrafish, mice, and chicks [6,7,8,9,10]. In the same way, an increased latency of PTZ-induced seizures was observed by the treatment administration of the hydroalcoholic extract (50, 100, 200, 500, and 1000 mg/kg) and aqueous and ethyl acetate fractions (25 and 100 mg/kg) demonstrating its anticonvulsant and neuroprotector effect of *C. sativum* [11,12,13].

Continuing in this area, we will enunciate its chemical constituents, and we will land on those that have demonstrated their effect in pre-clinical models, either behaviorally or on some modes and pathophysiological mechanisms associated with these diseases, and those with defined activities on neurotransmission systems (GABAergic, glutamatergic, serotonergic). Finally, the advantages offered by in silico analyses for the determination of biological activities and directing the metabolites isolated from coriander to therapeutic targets will be discussed, as well as the use of novel delivery systems such as nanoparticles for the design of potential medicines derived from standardized extracts of *Coriandrum sativum*.

## 2. Methodology of Literature Research

The information search for this review is carried out based on the objective of presenting *Coriandrum sativum* as a plant species useful in the treatment of various diseases of a neurological and psychiatric nature. For this, we used the search engine on the database platforms Scopus, Google Scholar, and Pub-Med, based on the combination of the following keywords: “coriander”, “*Coriandrum sativum*”, “psychiatric disorders”, “anxiety”, “depression”, “epilepsy”, “sleep disorders”, “mood disorders”, “nervous”, “neurotransmitters”, “GABA”, “glutamate”, “serotonin”, “neurotransmitter’s receptors”, “treatment”, “phytochemistry”, “nanotechnology”, and “molecular docking”. A timespan from 2015 to date was established in both original and review articles indistinctly of the content under the condition that keywords were included.

This project was approved by the research and ethics committee of the Mexican Institute of Social Security, which assigned it the registration number R-2020-1702-005.

## 3. Mental Disorders

The WHO defines suitable mental health as related to mental and psychological well-being [14].

### 3.1. Impact of Psychiatric Disorders on Society

The same organization, WHO, indicates that anxiety and depression disorders are common medical conditions among the world population, affecting work capacity and, with it, productivity, causing the loss of about a billion U.S. dollars per year. In 2017, it was estimated that more than 300 million people in the world suffer from depression, and more than 260 million live with anxiety disorders, whereas conditions such as epilepsy affect more than 50 million patients [15,16]. The association between mental disorders and insomnia is noteworthy, with a prevalence of comorbidity between 30 and 60%; even up to 80% of patients with depression suffer from insomnia. In addition, the persistence of insomnia is related to the development of some other mental disorders [17].

### 3.2. Anxiety Disorders (AD)

A certain degree of anxiety is normal and allows adaptation behaviors to different circumstances of the environment [18]. However, when reactions are exacerbated by apparently normal or even non-existent stimuli, they can be clinically diagnosed as anxiety disorders (AD) which are the most prevalent psychiatric disorders in the world, with a rate close to 7.3%. Specific phobias are the most common, with 10.3%, followed by panic disorder (6%), social phobia (2.7%), and generalized anxiety disorder (GAD) with 2.2% [19].

These conditions constitute a series of medical circumstances clinically differentiated. The guide for clinical diagnosis DSM-V (Diagnostic and Statistical Manual of Mental Disorders) [20] indicates two large groups, primary anxiety disorders, including panic disorders, agoraphobia, specific phobia, social anxiety disorder, selective mutism, GAD, separation anxiety disorder, anxiety disorder due to other medical condition, substance/drug-induced anxiety disorder, and other disorders, with noticeable anxiety symptoms that do not correspond to any of the above. The second group is cataloged as other causes of anxiety and related symptoms, which include: obsessive-compulsive disorder, post-traumatic stress disorder, acute stress disorder, avoidant personality disorder, somatic symptom disorder, and illness anxiety disorder [21].

ADs constitute the largest group of mental disorders in Western societies and are a cause of disability, which generates indirect costs in addition to the direct costs associated with treatment. These have an onset at an early age; if patients are not treated, the symptoms become recurrent and chronic, and anxiety symptoms are often followed by those associated with depression [22]. The characteristics of ADs are excessive and lasting fear, avoidance of perceived threats, real or not, which can be in social situations or bodily sensations, and patients present panic attacks in response to uncontrolled fear, the latter as a hallmark of these disorders [20]. Currently, it is considered that the etiological factors associated with the presence of AD include psychosocial, such as the experience of adversity during childhood and adolescence, stressful events throughout the history of individuals, and genetic vulnerability [19].

### 3.3. Mood Disorders: Emphasis on Depression

Based on the DSM-V, which uses some criteria for the diagnosis of mental problems related to mood, establishes that a “mood disorder” is a pattern of illness that derives from an altered mood. Almost all patients with one of these disorders experience depression at some point [20].

Depressive disorders are classified in this guide as major depressive disorder, persistent depressive disorder (dysthymia), disruptive mood dysregulation disorder, premenstrual dysphoric disorder, and bipolar disorder [21].

Major depressive disorder (MDD) is one of the most prevalent and highly disabling psychiatric disorders. Some of its symptoms include impaired cognition, motor and motivation impairment, decreased concentration, profound sadness, abnormal loss of interest and pleasure, changes in appetite and/or weight, sleep disturbances, agitation or lethargy, abnormal fatigue or loss of energy, self-reproach or inappropriate guilt, indecision, and morbid thoughts of death or suicide.

### 3.4. Sleep Disorders

Sleep is a fundamental process of the central nervous system (CNS), which occupies up to a third of human life. As such, it can be one of the most important psychophysiological processes for brain function and mental health; it is essential to maintain the physical and mental well-being of individuals. A disturbance of this physiological state reduces the quality of life and tranquility, being a risk factor for secondary diseases. It is a fundamental functional state of the CNS, which allows for the maintenance of psychophysiological aspects, brain activity, and mental health. The alteration of this mechanism has adverse effects on the general health of individuals in the emotional and interpersonal sphere, cognitive processes (consolidation and reorganization of memory), problem-solving and creativity, emotional empathy, and interpersonal conflict management [23].

Sleep disorders (SDs) are grouped in basically insomnia (trouble sleeping or staying asleep), hypersomnolence (excessive sleep), and sleep–wake circadian rhythm disorders, with further subdivisions among each category [20,21]. Nonetheless, insomnia is frequently a symptom that causes medical consultation and that only sometimes constitutes an independent diagnosis of a major mental disorder or some medical condition. On the other hand, the ICSD-3 (International Classification of Sleep Disorders) [24] designates insomnia, sleep-related breathing disorders, central hypersomnolence disorders, parasomnias, sleep-related movement disorders, and circadian rhythm sleep disorders as the main classification categories [25].

### 3.5. Epilepsy

The WHO indicates epilepsy as a chronic neurological disorder characterized by repeated seizures with a strong potential for recurrence. Seizures are brief episodes of involuntary movements that can affect part of the body (partial seizures) or the entire body (generalized seizures) and are sometimes accompanied by loss of consciousness and sphincter control. It affects people of all ages, with social, behavioral, economic, and health consequences, not only for patients but also for their caregivers. It is estimated that 70% of patients with treatment respond positively, and the other 30% are resistant [16]. However, even though there are affordable treatments for epilepsy, in low- and middle-income countries, up to 90% of epilepsy cases may be undiagnosed or inadequately treated [26,27].

The global burden of epilepsy is high, affecting 50 million people. It is proposed that the number of patients increases due to life expectancy and the percentage of people who survive traumatic injuries, brain infections, etc. Around the world, people with epilepsy and their families suffer stigmatization and discrimination, with problems in accessing education, employment, and socialization with a low quality of living [16].

## 4. Neurotransmitters Involved in Psychiatric Disorders

In addition to the emotional burden and disability that, per se, these diseases bring with them, we must also consider the comorbidity that is established between them, increasing the patient’s suffering, treatment costs, and the care of the patient either by family members or specialized personnel [23]. The comorbidity between the various mental disorders and other diseases could be due to an interaction between abnormalities of different neurotransmission systems and other pathophysiological processes, such as inflammation and oxidation, as presented below.

### 4.1. GABA and Glutamate

Two important amino acids are γ-aminobutyric acid (GABA) and glutamic acid (glutamate, GLU). Both act as neurotransmitters and regulate the balance in neuronal communication between areas. The former is the main mediator of the most abundant type of synapse, the inhibitory one [28]. In contrast, the second is responsible for modulating the excitatory synapse [29,30]. They are of utmost importance for correct neurotransmission, and their imbalance gives rise to various neurological and psychiatric diseases [31]. Each of them exerts its actions through its union with a gamma of different receptors.

For GABA, ionotropic receptors are classified as type A (GABA_A_R) and metabotropic type B (GABA_B_R). The first is widely characterized and forms a family of different pentameric complexes located post-synaptically. Different subtypes of this receptor are formed by a repertoire of at least 19 subunits (α1-6, β1-3, γ1-3, δ, ε, θ, π, ρ1-3). These molecular rearrangements give rise to a receptor with a central channel that is permeable to chloride ions. Many of these receptors contain the α, β, and γ subunits, including the α1β2γ2 receptor, which is the most abundant, constituting approximately 60% of all GABA_A_ receptors in the brain [32].

While GABA_B_ receptors modulate the generation of excitatory postsynaptic potentials and long-term potentiation, these are made up of two subunits, B1 and B2, each with seven transmembrane domains that join to form heterodimers in which both units are necessary to form a functional receptor. Ligands bind to B1 in the N-terminal domain extracellularly, and B2 is responsible for interacting with the corresponding G protein. Both GABA_A_ and GABA_B_ receptors are involved in pathological processes such as sleep disorders, epilepsy, anxiety, depression, and memory, among others [33].

GLU is a critical modulator of brain functions. It acts on various receptor proteins located both pre- and post-synaptically in almost all areas of the CNS. There are ionotropic receptors (iGluRs), which are multimeric ion channels responsible for rapid transmission. They include the N-methyl-D-aspartate (NMDAR) receptors, α-amino-3-hydroxy-5-methyl-4-isoxazole propionic acid (AMPAR, formerly quisqualate), and kainate. Metabotropic (mGluRs) mediates the slow transmission, with long-term changes in synaptic activity. These are divided into eight subtypes grouped into three categories based on their homology, signal transduction mechanism, and pharmacological profile. Thus, we find group I (mGluR1 and mGluR5), group II (mGluR2 and mGluR3), and group III (mGluR4, 6, 7, and 8) [30].

For the previous, substances capable of blocking glutamate output can be useful in treating anxiety.

### 4.2. Serotonin: 5-hydroxytryptamine (5-HT)

The neurotransmitter 5-hydroxytryptamine or serotonin is key in various physiological processes in the CNS. It is a monoamine that acts on the regulation of emotions, circadian rhythms (sleep–wake), and cognition. Such effects are the result of binding to a wide variety of receptor molecules, which belong to seven families, 5-HT1-7, that comprise at least 14 different receptor subtypes that are structurally different, responding to a variety of pharmacological stimuli. They are all coupled to G proteins, and different members of each family are expressed in the brain. Among the most studied CNS diseases, we find 5-HT1, which are coupled to Gi/o proteins, and of these, 5-HT1A and 5-HT1B function as somatodendritic auto-receptors in neurons of the raphe nucleus, although they are also expressed as hetero-receptors. Perhaps the most studied subtype is 5-HT2C for its effects on depression and anxiety. This molecule preferably couples with the Gq/11 protein and increases inositol phosphate and cytosolic Ca^2+^ concentrations. 5-HT2C receptors were identified in the hippocampus, amygdala, and substantia nigra, among other areas [34].

Although it has been shown that most serotonin receptors play a role in MDD, AD, and sleep disorders, the most studied to date are 5-HT1 and 5-HT2 [35,36]. To a lesser or greater extent, the various types of serotonergic receptors have been implicated in different mental disorders; for example, 5-HT3, which is a homomeric receptor, and its subunits consist of an N-terminal domain, four transmembrane domains (TM1 to TM4) and an intracellular domain. One peculiarity of this receptor is that it is the only one of all serotonin receptors that mediate rapid excitatory responses through a cation channel (sodium, potassium, and calcium) activated by this neurotransmitter. It has been implicated in mood disorders and drugs of abuse, also in cognition and anxiety, found primarily in the limbic system, brain stem, and spinal cord. Antagonists to this biomolecule have effects such as antidepressants, anticonvulsants (although their pro-convulsant effect is also indicated), and anxiolytics, but not as sedatives or hypnotics [37].

## 5. Inflammation and Oxidative Stress Associated with AD, MDD, TS, and Epilepsy

The immune system (IS) protects the organism from the aggressions they are exposed to by recognizing the causative agent (trauma or infection), containment the damage, and regulating the magnitude and duration of the immune response that can harm tissues. Inflammation results from the system activation by injury or infections, causing warmth, redness, swelling, pain, and fever to eliminate the insult. After activation, immune cells produce mediators such as cytokines as regulators of the inflammatory response. The brain has specialized immune cells, called microglia, that maintain homeostasis and provide a response to damage, such as in neurodegenerative and neuropsychiatric states [38].

Patients with anxiety and depression present inflammatory response with an increase in the serum concentration of pro-inflammatory cytokines, such as interleukins (IL)-6, tumor necrosis factor (TNF), IL-12, IL-13, and IL-18, between other mediators, including C-reactive protein (PC-R) [39,40].

On the other hand, epilepsy is associated with neuronal damage, gliosis, and microgliosis, with a persistent inflammatory state in neuronal tissue. The cytokines IL-Iβ, IL-2, and IL-6, found in low concentration in the brain, are significantly increased after seizures. In a clinical study, IL-1β, IL-6, and tumor necrosis factor-α (TNF-α) in cerebrospinal fluid were found to be increased in febrile seizures, and the expression of these same molecules was upregulated in the hippocampus [41,42].

Natural sleep has a dynamic role in the regulation of the immune system, disturbances in this physiological state influence the activation of immune cells and the production of pro-inflammatory cytokines. Partial nocturnal sleep deprivation activates inflammatory signaling pathways, such as nuclear factor-κappaB (NF-κB), activator protein 1 (AP-1), signal transducer and activators of transcription (STAT), leading to increased levels of mRNA encoding pro-inflammatory cytokines IL-6 and TNF [43]. One of the early pieces of evidence of the relationship between IS and sleep was published in 1989, where was reported that prolonged loss of hours of sleep; also, 40 h of wakefulness induced elevated levels of IL-1 and IL-2. This increase was independent of the circadian rhythm of cortisol [44].

Oxidative stress (OE) is an imbalance between oxidant and antioxidant factors in favor of a pro-oxidant state, which is part of the immune response mechanism to damage. Although the brain constitutes 2% of the total weight in humans, the organ demands the most oxygen, up to 20% at rest. It is specifically vulnerable to the harmful effects of oxidative stress due to its low capacity for division and cell repair for the metabolic characteristics of neurons make them more sensitive to oxidation than other cells [45].

There is evidence of a connection between OE and psychiatric and neurological disorders. For example, glutamate-mediated excitotoxicity is mentioned as one of the main causative events in the generation of EO in the brain. The over-activation of the hypothalamic–pituitary–adrenal (HPA) axis during ADs and MDD mediates the release of glucocorticoids, causing deregulation of the function of the receptors to these hormones, which leads to a state of OE by affecting the mitochondrial metabolism. Studies with rodents show that OE is a regulator of feedback in the HPA axis and can alter GABAergic and serotoninergic neurotransmission, both involved in psychiatric disorders. It has also been shown that inhibiting the synthesis of glutathione (GSH) by the intrahippocampal administration of the L-buthionine-(S, R) sulfoximine induces anxiety behavior. GSH is present in all mammalian cells, and among other functions, it is an important determinant in redox signaling and is the most critical nonenzymatic endogenous antioxidant [46].

For example, depression has been associated with low levels of natural antioxidants (enzymes such as catalase (CAT) and superoxide dismutase (SOD)) and vitamin E in the plasma [47]. Clinical evidence suggests that patients with anxiety disorders have elevated levels of oxidized compounds in red blood cells, mononuclear cells, urine, and brain-spinal fluid [48]. Moreover, the participation of OE in epilepsy is evident and is considered a possible mechanism in the pathophysiology of the disease. For example, during an epileptic seizure, the production of free radicals increases, and the level of ATP decreases, which leads to a deficiency in the production and energy supply in the brain [49]. Oxidative damage to proteins, lipids, and DNA has also been observed during status epilepticus; deterioration in the antioxidant capacity of GSH is associated with neuronal death, cognitive deficit, and mortality in epilepsy models [50].

A controversial hypothesis says that one of the functions of sleep is the purification of ROS that accumulates in neurons during waking; thus, sleep works as a brain antioxidant [51]. However, some research groups report that rats or mice deprived of have a decrease in the antioxidant capacity of the brain [52,53], other reports contradict the finding [54].

Recently, it was published that sleep loss alters the redox state of several sleep-regulating neurons in the fly brain, influencing their activity [55]. Several other studies do not find important data on oxidative damage in the brain, so they have focused their work on finding evidence that, during sleep disturbance, there is a decrease in antioxidant defenses in the liver and other visceral organs, such as the gut [56,57].

The role of both inflammation and OE, in the pathophysiology of neurological and psychiatric diseases, represent potential therapeutic targets, in the pharmacological search for new and better treatments that allow for raising the quality of life of patients. In such a way, the search for new molecules of natural and/or synthetic origin is an objective of the study in many areas of research in the pharmacology of ADs, MDD, sleep disorders, and epilepsy.

## 6. Treatments for AD, MDD, TS, and Epilepsy

**Figure 1:**
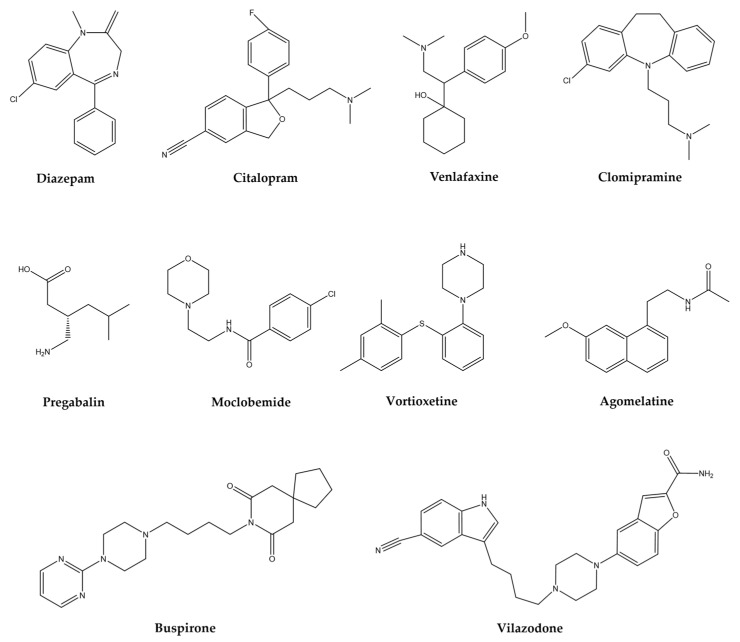
Chemical structures of the leading drugs for neurological and psychiatric disorders treatment.

The treatment of mental disorders, focused on in this review, share some elements, this association is because some brain areas involved in the pathophysiology of each mental disorder are commonly shared. The mechanism of action and clinical indication of the main pharmacological groups (Figure 1) prescribed for neurological and psychiatric disorders are summarized in Table 1.

The widely used substances such as anxiolytics, sedatives, and antidepressants, by excellence, are benzodiazepines (BZDs). They are allosteric modulators of the GABA_A_ receptor such as diazepam, clonazepam, alprazolam, chlordiazepoxide, clorazepate, lorazepam, among others [58]. The adverse effects reported for this group of substances are CNS depression that causes fatigue, dizziness, increased reaction time, impaired driving ability, and impaired cognitive function, especially in elderly people, as well as tolerance, dependence, and addiction, with rapid oral or parenteral action [59].

Another group of drugs used to treat these disorders includes selective serotonin reuptake inhibitors (SSRIs) among which stand out: citalopram, escitalopram, fluoxetine, fluvoxamine, paroxetine, and sertraline. The adverse effects described for this group are nervousness, nausea, restlessness, headache, fatigue, increased or decreased appetite, weight gain, weight loss, tremors, sweating, prolongation of the QTc wave in the electrocardiogram, sexual dysfunction, diarrhea, constipation [60,61].

In addition, the serotonin/norepinephrine (SNRIs) group: duloxetine and venlafaxine, with the following effects: nervousness, nausea, restlessness, headache, fatigue, increased or decreased appetite, weight gain, weight loss, tremors, sweating, sexual dysfunction, diarrhea, constipation, urination problems and other side effects [62]. These substances are recommended as first-line treatment, used for both anxiety and depression, and some of them have a latency of 2 to 4 weeks, although it can last up to 6 weeks, which is a factor that affects the patients’ detachment from such medication, coupled with the fact that during this period the undesirable symptoms associated with these drugs appears with greater intensity.

The tricyclic antidepressants (TCAs), non-selective serotonin reuptake inhibitors such as clomipramine or imipramine, exhibit adverse collateral symptoms that include: anticholinergic effects, drowsiness, dizziness, cardiovascular side effects, weight gain, nausea, headache, and sexual dysfunction, among others [63,64].

There exist many other drugs like pregabalin also prescribed for anxiety disorders, which is a calcium modulator, whose data on adverse symptoms are referred to as dizziness, drowsiness, dry mouth, edema, blurred vision, weight gain, constipation, euphoric mood balance disorder, increased appetite, difficulty concentrating/attention, and withdrawal symptoms after an abrupt medication stop [65,66]. Azapirones (AZA), like buspirone, reduce anxiety states and act on 5-HT-1A receptors for serotonin, associated problems are dizziness, nausea, headache, nervousness, lightheadedness, excitement, and insomnia [67]. Another drug is moclobemide whose mechanism of action is to reversibly inhibit the enzyme monoamine oxidase, thereby modifying the metabolism of norepinephrine, dopamine, and serotonin, increasing their availability in the synaptic cleft. These drugs also cause restlessness, insomnia, dry mouth, headache, dizziness, gastrointestinal symptoms, and nausea [68].

Some other synthetic drugs, novel due to their structure and mechanism of action, t proposed for the treatment of some psychiatric disorders such as anxiety and depression. Like vortioxetine, which is a compound that inhibits the serotonin transporter (SERT), in addition, to act as a full agonist of the 5-HT1A receptor, a partial agonist of 5-HT1b and antagonist of 5-HT1d, 5-HT3, and 5-HT7, it also modulates GABAergic and glutamatergic transmission; and in clinical trials, it has an effect in patients with generalized anxiety [69]. Vilazodone with similar action to that of buspirone, in addition to blocking SERT, has been proposed as an anxiolytic and antidepressant. Agomelatine, an MT1 and MT2 receptor agonist for melatonin and a 5-HT2C antagonist is proposed as an antidepressant and anxiolytic [70]. 

Due to the complications associated with mental disorders and the treatments used to control them, much of the pre-clinical pharmacological studies in the search for better alternatives focus on using medicinal plants as an object of study. Especially those whose characteristics in metabolite production make them unique, such as *C. sativum*, and plants of the same family that synthesize volatile compounds, like *Foeniculum vulgare* (fennel) and *Petroselinum crispum* (parsley), which different medicinal and pharmacological properties are also attributed [71,72]. The information presented in the following section emphasizes marking the described activities of a food and medicinal species widely distributed worldwide.

**Table 1:** Mechanism of action and clinical indication of the main pharmacological groups prescribed for psychiatric disorders.

Group	Drugs	Mechanism of Action	Clinically				Reference
			Anxiolytic	Antidepressant	Sedative	Antinociceptive	
BZD	Diazepam, clonazepam, alprazolam, chlordiazepoxide, clorazepate, lorazepam	GABAA receptor allosteric modulators	+	+	+	+	[58,59]
SSRIs	Citalopram, escitalopram, fluoxetine, fluvoxamine, paroxetine, sertraline	Selective serotonin reuptake inhibition	+	+	−	−	[60,61]
SNRIs	Duloxetine, venlafaxine	Serotonin and norepinephrine reuptake inhibition	+	+	−	+	[62]
TCAs	Clomipramine, imipramine, desipramine, nortriptyline	Reuptake of serotonin and norepinephrine blockage	−	+	−	+	[63,64]
CaM	Pregabalin	Decrease calcium influx	+	+	+	+	[65,66]
AZA	Buspirone	Acts on 5-HT-1A receptors for serotonin	+	−	−	−	[67,68]
MAOIs	Moclobemide	Reversible inhibitor of the enzyme MAO	+	+	−	−	[73]
SERTIs	Vortioxetine	Serotonin transporter inhibition	+	−	−	−	[69,70]
SERTIs	Vilazodone	SERT blocker; acts on 5-HT-1A receptors for serotonin	+	+	−	−	[70]
MMAs	Agomelatine	Melatonergic receptor agonist and 5-HT2C antagonist	+	+	−	−	[70]

BZD: benzodiazepines; SSRIs: selective serotonin reuptake inhibitors; SNRIs: serotonin and norepinephrine reuptake inhibitors; TCAs: tricyclic antidepressants; AZA: azapirones; MAOIs: monoamine oxidase inhibitors; SERTIs: serotonin transporter inhibitors; MMA: melatonin and melatonergic agonists.

## 7. *Coriandrum sativum* L.

The genus *Coriandrum* belongs to the Apiaceae family, the leading group of flowering plants (Angiosperms). This herb is “generally recognized as safe” (GRAS) as a food by the U.S. Food and Drug Administration (FDA). Coriander is a plant species with enormous culinary importance in Mexico, becoming a relevant agri-food product so that the country is the fourth-world producer of this crop. In 2017 it generated an income of 47.3 million U.S. dollars from exports of approximately 64,647 tons [74]. 

In 2019, 6983 Ha were planted with a production of 100,034 tons valued at MXN 486 million. This herb has been used for almost 3000 years and was introduced during the Spanish conquest of Mexican territory, resulting in widespread acceptance and consumption [75]. This led to its incorporation and acceptance as part of the rich mestizo gastronomy, which resulted from the Mesoamerican and European fusion with Mediterranean influence [76]. The first written reference to the culinary use of coriander dates from the 16th century in the following statement: “These moles (a Mexican recipe) are related to the pre-Hispanic pipians”, by Sevillian Juan de la Cueva (1543–1610) in his epistle dedicated to the lawyer Sánchez de Obregón, First Corregidor of Mexico; he mentioned a pre-Hispanic stew, which is still used in present-day preparations of a green mole or “pipian”. The miscegenation of this stew includes Mesoamerican food products such as turkey, epazote, pumpkin seed, peanut, chili peppers, and green tomatoes, which were fused with chard, lettuce, coriander, almonds, hazelnuts, sesame, pork, and garlic, brought by the Spanish [76].

Cultivating coriander allows the resource to be available throughout the year, providing the aroma that recalls that of fresh citrus peel, with a fine reminiscence of sage. All of the above is due to its rich composition of essential oils, highlighting linalool (74.6%), menthone (5.9%), geranyl acetate (4.6%), p-cymene (4.0%), and trans-geraniol (2.8%) (Samojlik et al., 2010). The same justifies the other inherent use as a therapeutic resource in Ayurvedic, Chinese, and Iranian medicine [77].

### 7.1. Botanical Description and Traditional Uses

It is an annual plant approximately 50 cm high, with leaves piled up in the lower part. They are split and have indentations, as well as a characteristic odor. Its flowers are white to lilac tones, slightly shaped like an umbrella, with spherical and small fruits. It lives in warm, semi-warm, and temperate climates between 1000 and 3000 m above sea level [4].

### 7.2. Plant Ethnobotany on CNS Diseases in Mexico

There are several medicinal uses attributed to coriander. However, for nervous disorders, its usefulness is referenced for weakness and fatigue, to promote sleep, and for “altered nerves”. Data from the 17th century indicate its use to reduce headaches and induce sleep, among others. Vicente Cervantes wrote at the end of the 18th century, “The seed is something narcotic and carminative”. In the 20th century, Maximino Martínez referred to the plant as a stimulant [4]. 

Popular Mexican synonym: Cilantro de zopilote, perejil; Michoacán: kurhantru (purhépecha); Puebla: zopiloxuitl (náhuatl); San Luis Potosí: kulantu’ (tenek).

The plant is classified as follows:

Kingdom: Plantae

Division: Magnoliophyta

Class: Magnoliopsida

Order: Apiales

  Family: Apiaceae

  Subfamily: Apioidea

  Tribe: Coriandreae

  Genus: Coriandrum

    Species: *Coriandrum sativum* L.

## 8. Phytochemical Constituents of *C. sativum* and Its Activities in Psychiatric Disorders

### 8.1. Essential Oils

The essential oil (EO) obtained from the seeds of *Coriandrum sativum* is widely used in traditional medicine and the food, pharmaceutical, and cosmetic industries [78,79]. Moreover, the essential oil from this plant has been reported to induce sedative effects in rodents, chickens, and fish, which can be related to the main components identified by mass-coupled gas chromatography (GC-MS) and gas chromatography with flame ionization detector (GC-FID) techniques. The effect is due to the high percentages of the most abundant compounds, such as linalool (64.8–81.7%), α-pinene (5.5–7.3%), and γ-terpinene (5.6–5.7%). In addition to the presence of other monoterpenes and alkanes, the following stand out: camphor (3.1 ± 0.4), β-ocimene (0.79 ± 0.02), limonene (0.7 ± 0.1), geranyl acetate (0.68 ± 0.08), myrcene (0.58 ± 0.05), camphene (0.43 ± 0.02), β-pinene (0.41 ± 0.05), dodecane (0.18 ± 0.02), and tetradecane (0.34 ± 0.02) [6,80].

In the EO of the fruits, linalool is also found as the main component, which varies in concentration from 68% to 82% due to the different stages of maturity used in each study. For example, in nuts, the presence of significant compounds such as linalool (82%), geraniol (6%), α-pinene (5%), β-pinene (3%), and thymol (1.48%) was determined, with the ability to reduce the duration and frequency of migraine attacks in a clinical study [5]. 

Another evaluation showed an improvement in cognitive function and anxiolytic and antidepressant effects. It is composed, in addition to linalool, of γ-terpinene (7.729%), α-pinene (6.509%), pinocarvone (4.388%), carvone (2.314%), β-ocimene (E + Z) (3.105%), and geranyl acetate (1.580%) [10].

In addition to the compounds previously presented, EOs contains others that belong to the family of oxygenated monoterpenes (77.8%), hydrocarbon sesquiterpenes (3.7%), and monoterpene hydrocarbons (17.6%), such as α-thujene, camphene, sabinene, β-pinene, myrcene, δ-3-carene, p-cymene, limonene, 1,8-cineole, cis-sabinene hydrate, cis-linalool oxide, terpinolene, camphor, borneol, terpinen-4-ol, p-cimen-8-ol α-terpineol, estragole, safranal, n-decanal, citronellol, neral, geraniol, geranial, thymol, 10-undecenal, myrtenol acetate, neryl acetate, (E)-2-undecenal, and (E)-Caryophyllene, which associates the lipophilic nature of EA with alterations in the plasma membrane [81].

### 8.2. Extracts and Active Compounds on Nervous Disorders

In the aqueous extract of seeds and aerial parts of *C. sativum*, the presence of micro- and macronutrients is reported to which activities this species has on the CNS are attributed to a certain extent, such as neuroprotective, anxiolytic, and antidepressant, among others. In addition, this extract is analgesic, and the authors mention that the activity is also due to the high concentration of linalool, which, as already mentioned, is extracted from EOs [82]. Another study indicates that exposure to this monoterpene in fish can cause sedative and analgesic activity [83].

An aqueous extract obtained from dried leaves confirmed its anxiolytic effect at doses of 50, 100, and 200 mg/kg. In an elevated plus-maze test, both the number of entries and the time spent in the open arms were significantly increased to values similar to those obtained by the drug diazepam in a dose-dependent manner. In the evaluation, it is considered that the presence of sterols, tannins, and flavonoids gives the extract its effect [8].

It is also mentioned that compounds of another nature, such as polyphenols, could add to this property since some compounds as caffeic acid and gallic acid, have been identified, along with steroids, coumarins, carotenoids, terpenes, and other components [82]. In an aqueous extract of stems and leaves, the presence of minerals such as calcium (11,024.5 ± 944 mg/mL), magnesium (3041.2 ± 121 mg/mL), copper (13.6 ± 0.173 mg/mL), Iron (110.7 ± 9.18 mg/mL), zinc (72.48 ± 1.21 mg/mL), and manganese (34.6 ± 0.174 mg/mL) was observed. These minerals individually have a role in neuromodulation, interaction with heavy metals, antioxidant enzyme synthesis, and biosynthesis of neurotransmitters [84].

The chemical characterization of the hydroalcoholic extract is based mainly on the quantification of the content of polyphenols and total flavonoids, whose neuroprotective effect is based on its antioxidant capacity, reducing the presence of psychiatric and cognitive problems such as depression, anxiety, and memory loss [13,85]. In a model in zebrafish, the fear and anxiety responses induced by exposure to alarm substances were evaluated, where the anxiogenic and alteration effects were prevented when the fish were injected with the hydroalcoholic extract of coriander at 50 and 100 mg/kg. Through high-performance chromatography analysis, the compounds gallic acid (1.88 µg/mL), caffeic acid (2.32 µg/mL), luteolin (5.61 µg/mL), quercetin 3-β-d-glucoside (43.56 µg/mL), and rutin (3.21 µg/mL) were identified [7].

Several factors affect the performance in obtaining bioactive compounds in an extraction method, so the reports on the content of total phenolic compounds, measured as mg of gallic acid equivalents/g of dry weight, varies between 66 and 820 mg GAE/g DW, where the maximum value corresponds to a hydroalcoholic extraction (Ethanol/Water; 50/50 *v*/*v*) assisted with microwaves [86,87]. At the same time, the total flavonoid content reported is 16.14 ± 1.17 mg of quercetin equivalents (QE)/g of dry extract, compared with 2.83 ± 0.20 mg QE/g DW for the stems, where both were able to exert anti-inflammatory activity on RAW 264.7 macrophages stimulated with LPS [88].

The hydroalcoholic extract of coriander stems and leaves and their fractions, obtained with solvents such as water, ethyl acetate, and butanol, contain flavonoids (3.70 ± 1.1 mg/100 g), flavonols (1.67 ± 0.5 mg/100 g), and tannins (2.80 ± 0.5 mg/100 g), and pharmacological activities such as anxiolytic, hypnotic, and neuroprotective have been detected using both in vivo and in vitro models of neurodegenerative diseases, seizure tests caused by pentylenetetrazole (PTZ), and sleep-potentiation evaluations with barbiturates [9,12]. Similar evaluations of the neuroprotective effects in PTZ-induced seizures in rats were carried out with the i.p. administration of ranging doses from 50 to 1000 mg/kg of a hydroalcoholic extract (70/30, *v*/*v*) by a Soxhlet apparatus. The extract, in addition to preventing the formation of dark neurons and apoptotic cells, was able to reduce the duration and increase the latency time of the attacks associated with the potential antioxidants, such as linalool, contained in the aerial parts of the plant [11,13].

In the methanolic extract of coriander, aldehydes, alcohols, and phenolic acids were identified as decanal, (E)-2-decenal, undecanal, (E)-2-undecenal, (E)-2-dodecanol, tri-decanal, phytol, vanillic acid, and 3,4-dihydroxybenzoic acid. The compound (E)-2-dodecenal turned out to be the molecular basis of coriander for activating KCNQ potassium channels, which increased the latency period in the outbreak of seizures [89].

In a hexane extract, compounds such as tripetroselinin, 1,3-dipretroselenin, petroselinic acid, and linalool were determined. The same authors identified tripetroselinin in the methanolic extract as the majority, in addition to 2-C-methyl-D-erythritol, 4-O-β-D-glucopyranoside, and sugars (fructose, glucose, sucrose, and nigellamosa), identifying that some of these substances exert a potent inhibition of lipid peroxidation [90].

The concentration of saturated, polyunsaturated, and monounsaturated fatty acids was established with values of 4.42%, 15.57%, and 79.794%, respectively, in an extract obtained from chloroform and methanol with high antioxidant effects. These values represent 99.93% of the extract. The rest corresponds to the content of phenolic compounds and sterols such as β-sitosterol (997.967 mg/kg), cycloartenol (51.29 mg/kg), and Δ7-avenasterol [91].

Using GCMS, the ethyl acetate fractions (F-AcOEt) obtained from the dichloromethane extract of the aerial parts were evaluated to determine their antinociceptive properties. In the dichloromethane extract, the following constituents were identified: n-hexadecanoic acid (6.91%), α-linoleic acid (12.58%), stigmasterol (7.04%), phytol (7.01%), and α-sitosterol (0.99%), as well as two isocoumarins: coriandrine (5.37%) and dihydrochoriandrine (15.64%). In contrast, in the F-AcOEt, compounds such as furanocoumarins were identified: dihydrocoriandrine (34.5%), choriandrine (14.4%), vitamin E (4.6%), and stigmasterol (7.9%) [92].

It should be noted that *C. sativum* is a rich source of folates, which have an essential role in avoiding nutritional disorders, primarily physiological and psychological problems. Recently, an elicitation by salicylic acid and the evaluation with HPLC of microwave-assisted extraction of its leaves showed different sources of folate together with their concentrations, which are presented below: 5-CH3-H4 folate (853 ± 22.8 µg/100 g DW) as the primary form, followed by folate 5-HCO-H4 (519 ± 24.0 µg/100 g DW), and in smaller amounts, folate 10-HCO-H4 (59.3 ± 4.66 µg/100 g DW), with a total content of approximately 1330 µg/100 g DW [93].

Also, different techniques have detected and extracted flavonoids; for example, quercetin, isoquercitrin, rutin, pinocembrin, apigenin, pseudobaptigenin, galanin-5-methyl ether, baicalein trimethyl ether, kaempferol dimethyl ether, pinobanksin-5-methyl ether-3-O-acetate, and pinobanksin-3-O-pentanoate have been reported, along with pinobanksin-3-O-phenylpropionate, pinobanksin-3-O-pentanoate, apigenin-7-O-glucuronoid, quercetin-3-O-glucoside, apigenin-3-O-rutinoside, isorhamnetin-3-O-rutinoside, and quercetin dimethyl ether-3-O-rutinoside. In addition, daidzein, luteolin, pectolinarigenin, apigenin-C-glucoside, kaempferol-3-7-dimethyl ether-3-O-glucoside, and apigenin-7-O- (6-methyl-beta-D-glucoside) have also been reported [84,94,95,96].

## 9. Activities of Compounds from *C. sativum* over Mental Disorders

Some compounds isolated and identified in *C. sativum* have proven activity in nervous central system disorders. In addition, their interaction with some neurotransmission systems involved in various psychiatric and neurological medical conditions has even been demonstrated and summarized in Table 2. In this section, we will mention those compounds that have been evaluated in some pharmacological models and that make their effect as antidepressants, anxiolytics, anticonvulsants, or sedatives evident.

### 9.1. Essential Oil Terpenes

#### 9.1.1. Linalool

Although the composition of the EOs of *C. sativum* gives its pharmacological capacity on the CNS, this effect relies on its high concentration, and (−)-linalool could be the main responsible for such effect. This monoterpene is capable of interacting with different neurotransmission systems. For example, the 350 mg/kg dose in mice significantly blocks seizures (including myoclonic jerks, circling behavior, mild salivation, and tail biting) induced by N-methyl-D-aspartate, the NMDAR receptor agonist for glutamate [97]. In addition, the intraperitoneal administration of this compound exerts an antidepressant effect, at doses of 100 and 200 mg/kg, on mice exposed to the tail suspension test (TST) [98]. It was evidenced that this action is carried out by interacting with the serotonergic system through the 5-HT1A receptors. The same is reported for the compound and β-pinene [99].

#### 9.1.2. Borneol

The administration of borneol at 800 mg/kg exerts an anti-conflict effect in the Geller conflict tests, which indicates a probable anxiolytic effect [100]. In addition, this compound can reduce anxiety-related behavior, an effect blocked by the administration of bicuculline, a GABAA receptor antagonist [101].

The anticonvulsant activity of the bicyclic monoterpene was evaluated in the kindling model induced by PTZ (35 mg/kg), and the intraperitoneal application of borneol at 5, 10, and 25 mg/kg was compared with diazepam (1 mg/kg). A significant decrease in the epileptogenic process was observed [102].

#### 9.1.3. Camphene

An effect similar to that of borneol was observed with this compound in the Geller conflict test [100].

#### 9.1.4. δ-3-Carene

Administering this monoterpene orally at 12, 25, 50, and 100 mg/kg before pentobarbital (45 mg/kg) causes a sleep-enhancing effect induced by the barbiturate in a dose-like dependent manner; flumazenil blocks this effect. The same authors demonstrate that this compound in rat hippocampal brain slices can prolong the constant decay of spontaneous inhibitory postsynaptic currents (sIPSCs) in a dose-dependent manner. Again, this behavior was antagonized with flumazenil. Both studies indicate the possible interaction of 3-carene on the benzodiazepine receptors in GABAA, which was confirmed by molecular docking studies [103].

#### 9.1.5. Carvone

Natural terpene that can be purified as (S)-(+)-carvone and its enantiomer (R)-(−)-carvone. Both have been shown to decrease ambulation and are sedative by enhancing sleep induced by pentobarbital at 100 mg/kg, the latter being more effective. At doses of 200 mg/kg (S)-(+)-carvone increases the latency of the first seizure induced by pentylenetetrazole and picrotoxin; however, the (R) isomer was not active in these tests [104]. Subsequently, it was demonstrated in the primary culture of mouse neurons that both isomers inhibit the binding of [(3) H] flunitrazepam at the GABAA receptor. This suggests an interaction with this neurotransmission system. However, this study was conducted to explain the insecticidal effect of carvone [105], but it may also explain the sedative effect already mentioned.

#### 9.1.6. Geraniol

In rats, the intraperitoneal administration of this compound at doses of 25, 50, and 100 mg/kg causes CNS depressant effects. A decrease in motor activity was observed in the hole-board test and OFT. Furthermore, 200 mg/kg significantly increases the latency of the first seizure induced by pentylenetetrazol (competitive antagonist of GABAA) and protects against seizures in 50% of the animals. Finally, the researchers showed the capacity of 100 mg/kg of this compound to increase the sleep time induced by thiopental (GABAA agonist). They propose a possible mechanism for this central transmission mechanism [106]. Previously, it had already been shown that this molecule effectively blocks human 5-HT3A receptors expressed in *Xenopus laevis* oocytes [107].

#### 9.1.7. Limonene

The following doses of 25, 50, and 75 mg/kg administered intraperitoneally have a sedative effect on mice exposed to the OFT test. In EPM, oral and inhalation administration has been shown to have an anxiolytic effect in rodents. Both effects were blocked by flumazenil, so limonene likely acts on the GABAergic neurotransmission system [108,109]. The limonene inhalation method in mice with unpredictable chronic stress (CUMS) caused an antidepressant effect on FST [110]. 

#### 9.1.8. β-Myrcene

It is another constituent of *C. sativum* EOs, which produces a significant increase in pentobarbital-induced sleep time when administered orally in rats, acutely at 1.0 mg/kg. However, daily administration for 14 days (1.0 mg/kg) reduces this parameter. The authors indicate that it is a compound that interferes with the metabolism of barbiturates by activating the cytochrome P-450 enzyme [111]. Later studies indicate that the intraperitoneal administration of β-myrcene to mice (100 and 200 mg/kg) exerts a sedative effect by reducing the number of crosses, rearing, and grooming in the OFT. It also affects motor coordination (relaxing effect) and enhances the hypnotic effect of pentobarbital when a higher dose is applied. There is no anxiolytic effect in EPM [112]. The anticonvulsant activity of this terpene was evaluated with 200 mg/kg intraperitoneally in mice, using pentylenetetrazol, and an increase in the latency of the first seizure, as well as the survival percentage, was observed; the effect was also observed by oral administration, however, until using 400 mg/kg [113].

#### 9.1.9. α- and β-Pinene

Both monoterpenes are enantiomers that have been evaluated in different models of CNS activity. For example, in rats, by electroencephalography and electromyography studies, the hypnotic capacity of α-pinene was measured upon injection of 45 mg/kg of pentobarbital, and in molecular docking, it was observed that the effect is associated with GABAA receptors at the site of benzodiazepine binding [114]. Inhalation exposure to this terpene in rats causes an anxiolytic effect on EPM [115]. It was observed that α-pinene at 0.2 and 0.4 mg/kg reduces the mortality rate of animals with PTZ-induced seizures [116]. In addition, rats that had received a dose of 80 mg/kg of PTZ, and with the treatment of α-pinene, β-pinene, and a mixture of both, caused a significant decrease in the intensity of the seizures and an increase in the latency of the first convulsive event, especially with the 400 mg/kg dose of the mixture [117].

#### 9.1.10. γ-Terminene

A dose in mice of 0.3 mg/box (0.03 mg/L) using the inhalation method reduces spontaneous motor activity in OFT, suggesting a sedative effect, which does not appear to be due to motor incoordination. This compound enhances the effects of pentobarbital to induce sleep, and although it could affect benzodiazepine receptors, the authors point out that more experiments are needed to clarify it [118].

#### 9.1.11. γ-Terpinene

Also, when given by inhalation at 0.3 mg/box, it reduces spontaneous motor behavior. In the OFT, the pentobarbital potentiation test shows a behavior similar to diazepam, so it is concluded that it acts as a sedative without affecting motor coordination [118].

#### 9.1.12. Terpinen-4-ol

This volatile compound has been shown to exert a sedative effect, after exposure to mice, through inhalation, after which it causes a decrease in motor behavior [119]. The in vitro activity assay of the enzyme monoamine oxidase (MAO), as a measure of antidepressant activity, was carried out using 125 µg/mL of this monoterpene, and a percentage of inhibition of the enzyme of 42.5% was observed [120]. Different doses of terpinen-4-ol administered intraperitoneally were used to evaluate its neuropharmacological effect; thus, the 200 mg/kg dose decreased the motor activity of the mice and was able to enhance the sleep induced by pentobarbital. This same dose, in addition to that of 100 and 300 mg/kg, increased the latency of the first seizure similarly as diazepam does in the trial with pentylenetetrazol; however, in picrotoxin seizures, only the two highest were effective, while for maximum electroshock, the only one that decreased the presence of seizures was the greatest. Because pro-convulsants such as pentylenetetrazol and picrotoxin, and on the other hand, the sedative pentobarbital, modulate GABAergic transmission, the authors suspect that terpinen-4-ol could act at this level [104]. In a later study, the anticonvulsant effect of this molecule was confirmed in rats using pentylenetetrazole systemically, as well as by intracerebroventricular administration. The seizures induced by 3-mercaptopropionic acid were also lower and decreased sodium currents mediated by voltage-gated channels in electrophysiological recordings of the dorsal root ganglia. The administration of flumazenil did not antagonize the anticonvulsant activity, so it is concluded that the benzodiazepine receptor is not involved but that it may be modulating sodium channels [121].

#### 9.1.13. Thymol

This other terpene of *C. sativum* EOs and its isomer carvacrol activate the currents mediated by the channel of this molecule, acting as weak partial agonists and positive modulators of the same 5-HT3A receptor mentioned above. Thymol, at high concentrations, directly activates GABAA receptors, while at low concentrations, it is a positive allosteric modulator [107]. Thymol significantly decreases the spontaneous motor activity of mice when it is administered by inhalation at a dose of 0.3 mg/box [118].

### 9.2. Flavonoids

#### 9.2.1. Apigenin

It is capable of displacing flunitrazepan from its GABAergic receptor and exerting an anxiolytic effect at the 3 mg/kg dose in mice during the EPM test. The hole-board and locomotor activity tests show, at high doses (100 mg/kg), a sedative activity. This compound does not show anticonvulsant activity against PTZ when it is administered at 50 and 80 mg/kg [122].

Later, similar studies showed that apigenin injected into rats at 25 mg/kg reduces locomotor activity. In the same report, the authors found no anxiolytic activity at the 1 mg/kg dose. The researchers conclude that the sedative effect is not mediated by its action on benzodiazepine receptors because flumazenil does not counteract it [123].

Part of the mode of action by which the compound could exert its effects is through GABAergic modulation, for example, an in vitro model of the expression of the recombinant human GABAA receptor α1β2γ2L expressed in *Xenopus laevis* oocytes. That this molecule acts as a GABA antagonist on flumazenil-insensitive α1β2γ receptors indicates that it is not a negative modulator of the benzodiazepine site on the GABAA receptor [124].

Using the patch-clamp technique, it was observed that apigenin reversibly reduces the incoming currents produced by GABA. These effects do not explain the anxiolytic or sedative effect. In the same analysis, it was shown that the amplitude and frequency of spontaneous postsynaptic inhibitory currents (sIPSCs) on the α1β2γ2 receptor that was expressed in HEK293 cells also decreased in addition to blocking the responses induced by the NMDA receptor and inhibiting the amplitude and frequency of spontaneous postsynaptic excitatory currents (sEPSCs) in such a way that it acts as an antagonist of both types of channels (GABA and NMDA) [125]. This flavonoid also has an antidepressant effect, as indicated by Nakazawa et al. [126], who evaluated different doses in mice exposed to the FST test. They observed a decrease in immobility at the doses of 112.5 and 25 mg/kg treatments. Above these, they have no activity, which indicates a U-shaped curve, in addition to the fact that this activity does not modify the turnover rate of 5-HT. However, it does modify dopamine [126]. In a chronic model of depression induced by corticosterone administration (21 days), treatment with apigenin at 20 and 40 mg/kg reverses the reduction in “sucrose preference”. It also increases the immobility time in FST and reduces the alterations that in the levels of 5-HT in mice that are subjected to medium chronic stress (CMS) [127,128,129].

#### 9.2.2. Daidzein

The addition of this isoflavone (200 mg/kg) to the diet of mice for 30 days causes an anxiolytic effect when the individuals are placed in the EPM [129].

#### 9.2.3. Isoquercetin

It enhances the hypnotic effect of pentobarbital (50 mg/kg) after its intraperitoneal administration, increasing the duration of sleep in a dose-dependent manner (10, 25, and 50 mg/kg) [130]. When administered orally at 0.6 mg/kg in mice, it induces an antidepressant effect in the FST test. When the same dose is administered for 12 days, the effect is lost [131].

#### 9.2.4. Luteolin

A dose of 50 mg/kg in rats has been shown to produce an anxiolytic effect on EPM, and this behavior was not blocked by flumazenil at 3 mg/kg, so it was assumed that it is not dependent on receptor modulation of benzodiazepines [132]. Coleta et al. [133] showed the anxiolytic effect on EPM and antidepressant in FST with luteolin (5 mg/kg) in mice in addition to enhancing sleep induced by pentobarbital (1, 5, and 10 mg/kg). In this work, they determined the ability of this flavone to displace [3H] flunitrazepam from its binding site with a low affinity for benzodiazepine receptors. Both studies indicate that this compound exerts actions at the central nervous system level without necessarily being associated with this receptor [133]. In a model of stress induced by chronic administration of corticosterone in ICR mice, it was shown that oral administration of luteolin at 50 mg/kg/day/20 days induces a decrease in immobility time in the FST and TST, indicating an antidepressant response [134].

#### 9.2.5. Pinocembrin

Mice subjected to the chronic unpredictable mild stress model of depression received 10 mg/kg of this flavonoid for 20 days; in the end, they were exposed to different behavioral tests, including FST and TST. Animals treated with pinocembrin decreased their immobility behavior, indicating its antidepressant effect, in addition to restoring the motor behavior of the animals in OFT [135].

#### 9.2.6. Quercetin

This compound was administered to mice with mild traumatic brain injury at a dose of 50 mg/kg for 14 days; the mice showed a decrease in anxiety behavior in the OFT, EPM, LDB, and zero maze tests, but also the levels of corticosterone and adrenocorticotropic hormone, they were smaller than the animals that did not receive such treatment, which indicates the anxiolytic activity of the compound [136]. The intraperitoneal administration of quercetin in Wistar rats at a dose of 20 mg/kg/14 days also exerts antidepressant activity (FST), anxiolytic (EPM and LDB) and improves memory (MWM) [137]. The neuropharmacological effects of quercetin include anxiolytic and antidepressant, also effects on sleep, sedative, and anticonvulsant. This last effect seems to depend on the model used.

Thus, for example, it was shown that the daily intraperitoneal administration of quercetin (25 and 50 mg/kg) in rats in a kindling model induced by PTZ only exerts a slight anticonvulsant effect [138]. In contrast, the doses of 50 and 100 mg/kg in mice administered with kainic acid (glutamate receptor agonist) decreased seizure behavior and the expression of GABAA α5 receptors [139]. The different effects of quercetin on seizure threshold may occur through several mechanisms.

It has been shown, independently, that quercetin participates in the modulation of other mechanisms associated with TAs and TDs, for example, via serotonergic signaling. In addition, this flavonoid can inhibit the ionic current on the 5-HT3 receptor, in *Xenopus* oocytes, at an IC_50_ = 64 µM, in a reversible, concentration-dependent, and voltage-independent manner, acting as some other antagonists of this protein. The authors of these results mention that this natural molecule could be helpful in the prevention of nausea and vomiting induced by anticancer agents [140]; however, these data may indicate a mechanism of action associated with its effects on the aforementioned mental disorders.

#### 9.2.7. Rutin

In 2002, it was shown that the methanolic extract of *Hypericum perforatum* enriched with 9 mg/kg of rutin decreased the immobility time in FST. The flavonoid does not have antidepressant activity, but it is essential to increase the effectiveness of the treatment [141]. High doses of this compound, 300 to 1000 mg/kg, administered intraperitoneally to rats induce an increase in the number of entries and the time spent in the open arms of the EPM, denoting an anxiolytic effect that is partially antagonized by picrotoxin (blocks the GABAA chloride channel), but not by flumazenil (a benzodiazepine antagonist at the same receptor), so it is concluded that part of its mode of action is the interaction with the GABAergic system, without affecting the site of benzodiazepines [142]. In another study where rutin was administered for 16 days (30 mg/kg, 60 mg/kg, and 120 mg/kg), a reduction in immobility time was observed in the TST in addition to showing a statistical difference in the number of crosses in OFT [143]. Several studies confirm the antidepressant effect of rutin. Oral administration of 1 and up to 8 mg/kg are active in a dose-dependent manner in the TST test [144], while other data indicate that doses of 0.3 to 3.0 mg/kg are antidepressants in this test but not in FST in addition to not modifying motor activity in OFT. In this latter study, they found that the compound increases the availability of serotonin in the synaptic cleft [145]. Rutin (50 and 100 mg/kg) also attenuates the severity of seizures and improves memory in mice subjected to kindled with pentylenetetrazol [146].

**Table 2:** Bioactivities of isolated compounds from *Coriandrum sativum* by interaction with some neurotransmission systems involved in psychiatric and neurological disorders.

Phytochemical Group	Compound	Effect	Mechanism of Action	Reference
Terpenes	Borneol	Anticonvulsant and anxiolytic	GABA_A_R agonist	[100,101]
	Camphene	Anxiolytic	GABA_A_R agonist	[100]
	δ-3-carene	Sleep-enhancing	Interaction with BZD receptors in GABA_A_R	[103]
	Carvone	Delay of induced seizure Sleep-enhancing	GABA_A_R agonist	[104]
	Geraniol	Delay of induced seizure Sedative and sleep-enhancing	GABA_A_R agonist Blocks 5-HT3A receptors	[106,107]
	Limonene	Antidepressant, anxiolytic and sedative	GABA_A_R agonist	[108,109,110]
	(−)-Linalool	Anticonvulsant and antidepressant	GluR interaction through 5-HT1A receptors	[97,99]
	β-Myrcene	Anticonvulsant, sedative, and sleep-enhancing	Metabolism interference of barbiturates by cytochrome P-450 enzyme activation	[111,112,113]
	α- and β- pinene	Anxiolytic and hypnotic Delay of induced seizure	Interaction in BZD receptors in GABA_A_R	[114,117]
	γ-Terminene	Sedative and sleep-enhancing	Interaction in BZD receptors in GABA_A_R	[118]
	γ-Terpinene	Sedative effect	Similar behavior as diazepam	[118]
	Thymol	Sedative effect	Partial agonist and positive modulator of 5-HT3A receptor	[118]
	Terpinen-4-ol	Antidepressant, sedative, sleep-enhancing Delay of induced seizure	GABAergic transmission modulation without involving BZD receptors	[104,119,120,121]
Flavonoids	Apigenin	Anticonvulsant, antidepressant, anxiolytic, and sedative	GABAergic transmission modulation without involving BZD receptors Reduces alterations on the levels of 5-HT	[122,123,124,125,126,128,129]
	Daidzein	Anxiolytic effect	---	[129]
Flavonoids	Isoquercetin	Antidepressant and sleep-enhancing	----	[130,131]
	Luteolin	Antidepressant, anxiolytic, and sleep-enhancing	GABAergic transmission modulation without involving BZD receptors	[133,134]
	Pinocembrin	Antidepressant effect	----	[135]
	Quercetin	Anticonvulsant, antidepressant, anxiolytic, sleep-enhancing Memory improvement	Modulation of Glu receptor GABA_A_ α5 receptors expression modulation Ionic inhibition current on 5-HT3R	[136,137,138,139,140]
	Rutin	Antidepressant, anxiolytic, and seizures severity attenuation Memory improvement	GABAergic transmission modulation without involving BZD receptors GABA_A_ chloride channel blocking Serotonin availability increase in the synaptic cleft	[141,142,143,145,146]

**Figure 2:**
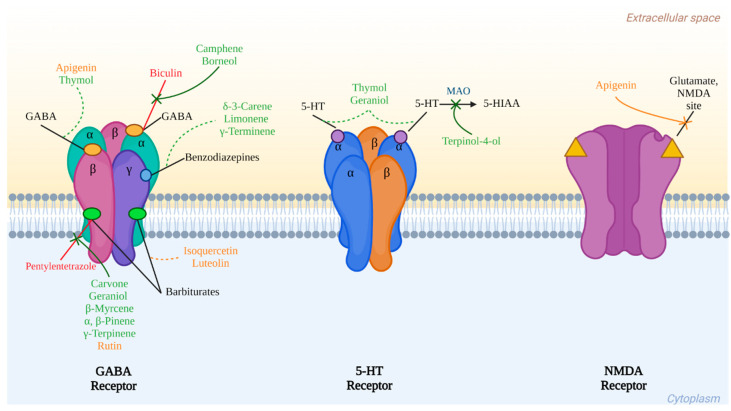
Summary of the drug interaction of active metabolites found in the medicinal and edible species *Coriandrum sativum*. Based on the data mentioned in the literature, this image summarizes the interaction of some flavonoid-like compounds (in orange) and terpenes (in green) with different types of receptors (GABA, serotonin, glutamate), which regulate neuronal function and are involved in neurological and psychiatric disorders that affect humans. In this way, it is proposed that coriander can become a phytomedicine formulated in nanoparticles whose utility can be for the treatment of psychiatric and neurological disorders that affect a suitable part of the world population. GABA receptor: receptors for the neurotransmitter γ-aminobutyric acid; 5-HT receptor: receptors for the neurotransmitter 5-hydroxytryptamine (serotonin); NMDA receptor: receptor type N-methyl-D-aspartate (ionotropic for the neurotransmitter glutamate).

The previously mentioned compounds that have been identified or isolated from cilantro and that present important biological activities in vitro and in vivo models related to central nervous system disorders summarize their interaction with three main receptors: GABA, serotonin, and glutamate, as is shown in Figure 2.

## 10. Metabolic Richness of *C. sativum* for the Design of Future Phytomedicine Based on Nanoparticles and Molecular Coupling Technologies

The content of metabolites that provide a high nutritional and medicinal value leads us to consider a future phytomedicine based on a standardized extract of *C. sativum* for treating neurological diseases such as those mentioned above based on nanoparticle technology and molecular analysis by docking technology. The proposal arises from the studies that have been made of this species in this line of technological research.

### 10.1. Molecular Docking

The association with proteins of some compounds, particularly flavonoids such as rutin, is analyzed by molecular docking as an approach to these compounds’ mechanism of action on a particular pathophysiological phenomenon. Table 3 summarizes those works that have been performed with *C. sativum* and whose design includes the analysis by molecular docking of compounds that this species contains. For example, an in vitro leukemia study with an extract rich in polyphenols obtained from coriander seeds was analyzed where the in vitro antitumor activity against cell lines K562 and HL60 was demonstrated, with an IC_50_ = 16.86 µM and 11.75 µM, respectively. This proposal measured the affinity of the main secondary metabolites on different leukemia-associated receptors, such as ABL kinase, ABL1, Bcl2, and FLT3 [147].

Flavonoids are secondary metabolites from this species tested by molecular docking as compounds that attenuate pathogenic markers of diabetic complications signals. These metabolites have an affinity for markers like advanced glycation end products (AGEs), sorbitol, and aldose reductase [150]. Between these compounds, quercetin, kaempferol, rutin, and the hydroalcoholic extract of *C. sativum* showed protective effects against streptozotocin-induced diabetic neuropathy in rats with anti-obesity and anti-nociceptive actions, decreasing lipid peroxidation in the sciatic nerve of diabetic rats and enhancing the levels of superoxide dismutase and glutathione. Also, the extract modulated AGEs, TNF-α, and nitrites levels. Attributing these pharmacological effects to the presence of metabolites, among which the flavonoids stand out, they were used to analyze the possible mode of action on TNF-α, a key modulator in the pathogenesis of diabetic neuropathy, with a suitable level of affinity [148].

In a similar study, the oil extract of this plant species effectively countered the damage caused by streptozotocin on weight, blood glucose, insulin, and serum lipid levels, decreased liver and kidney damage, inhibited AGEs, and had an antioxidant effect. AGE has been associated with the complications of diabetes mellitus because high glucose levels induce an increase in these products damaging organs and tissues, promoting this molecule toward a therapeutic approach. In this work, the authors then took linalool to a molecular docking study, coupling it to RAGE, a receptor element for AGEs, observing a suitable interaction with amino acid residues GLY200 and GLY199, VAL197 and VAL229, ASP201, ALP135 with a distance less than 2.45 Å [149].

In previous chapters, the anticonvulsant capacity of *C. sativum* is mentioned by activating KCNQ-type potassium channels. The main component that exerts this activity is fatty aldehyde (E)-2-dodecenal, capable of activating the channel on a site detected by molecular docking, juxtaposed between residues in the KCNQ S5 transmembrane segment and the S4-5 linker [89]. 

### 10.2. Nanoparticles

Nanoparticle technology is an essential tool that allows us to advance our knowledge of the pharmacology of known and new treatments, such as improving their bioavailability, efficacy, and safety. Nanotechnology has implicit multidisciplinary in the integration of various areas such as biology, engineering, chemistry, and physics [151,152].

This technological advance has been used to generate products that help the better development of *C. sativum* crops as a nutraceutical treatment for the protection of the species against toxic agents such as lead, improve the physiological characteristics of individuals, and improve their nutritional qualities, among others [153,154,155].

However, there is also research in the construction of nanoparticles of different materials, which aim to be the vehicle in which extracts of *C. sativum* can be evaluated for their medicinal properties; some examples are mentioned below.

Gold nanoparticles loaded with an extract of *C. sativum*, with a high content of anthocyanins, benzophenones, flavonoids, and phenols, whose antioxidant properties are mentioned in the literature. Jiao et al. evaluated the antioxidant capacity in vitro using the methods of ABTS radical scavenging activity, DPPH radical scavenging activity, and its analgesic effect by using paw licking in mice. The results showed a concentration-dependent antioxidant effect. Furthermore, the analgesic activity at a dose of 200 mg/kg of gold nanoparticles loaded with *C. sativum* was more significant than the administration of aspirin and the extract alone [152].

In another study, the synthesis of silver nanoparticles using an extract of *C. sativum* leaves was evaluated in vitro against acne (Propionibacterium acnes bacteria MTCC 1951) and dandruff (Malassezia furfur MTCC 1374). Inhibition on the growth of both microorganisms was observed with a minimum inhibitory concentration of 3.1 and 25 μg/mL, respectively. Also, a cytotoxic evaluation on mammary adenocarcinoma cell lines (MCF-7) showed an effect on the carcinogenic line, with an IC_50_ = 30.5 μg/mL, observing a complete inhibition at 100 μg/mL [156].

In experimental work, a design of lipid nanoparticles loaded with coriander essential oil was made, which contains high concentrations of compounds used in cosmetics, such as collagenase, elastase, and monoterpenes, such as linalool (81.29%). These were evaluated in vivo on skin exposed to UV rays. The dosage of such formulation of nanoparticles caused an increase in the content of collagen in the skin, as well as reduced levels of malondialdehyde (MDA), cyclooxygenase 2 (COX-2), prostaglandin E2 (PGE2), metalloproteinase-1 (MMP-1), Janus kinase (JNK), and activator protein-1 (AP-1), compared with the injured and untreated group [157].

Pharmaceutical technology is based on the formulation of nanoparticles loaded with coriander, a species with a wide range of biological activities and whose metabolic content suggests its usefulness in treating CNS diseases. Therefore, the approach of a line of research that leads to the development of a phytomedicine based on nanoparticles with actions such as anxiolytic, antidepressant, and anticonvulsant could be proposed.

## 11. Discussion

Mental health represents one of the significant challenges of today’s society. Although many neurological and psychiatric diseases are treated with available drugs, the task is not finished yet. In this way, it is still valid the search for alternatives that promote a better quality of life for people [14].

A physiopathological pivot for CNS diseases is the alteration in neurotransmission, then the disruption of GABAergic, glutamatergic, or serotonergic mechanisms, and the accumulation of damage resulting in chronic inflammation and oxidative stress events, all of which are associated factors in the development of mental illnesses [23]. Hence, many current treatments have the restoration or improvement of neurological systems as their therapeutic target, so *Coriandrum sativum* could act as a damage modulator on different pharmacological targets and effectively treat these diseases.

Therefore, investigating medicinal plants as producers of secondary metabolites of pharmacological utility is a feasible method to find, design, and propose phytomedicines. *Coriandrum sativum* is not only an edible plant widely used in the world, but it also represents an opportunity to take advantage of it as a pharmacological treatment for different diseases, among which those that affect the CNS can be pointed out [4,77]. The metabolic richness produced by this herb allows us to assume that in addition to the essential oils, other groups of compounds have been individually evaluated and have been shown to have biological actions related to pathophysiological processes involved in ADs, MDD, sleep disturbances, and epilepsy.

The present review considers the scientific elements of the research of Coriandrum sativum in the area of pharmacology, chemistry, technology based on molecular docking, and formulations with nanoparticles. Although data on the pre-clinical studies of coriander on models of CNS diseases are limited only to extracts, it should be noted that each of the elements included here puts into perspective that this species can become a phytomedicine with a clinical utility to alleviate mental disorders [11,12,13]. Its secondary metabolites can interact with the neurotransmission systems through its receptors, and other targets are subject to the activities of C. sativum as other neurotransmitters involved in other neurological and psychiatric diseases. In turn, its usefulness as an anti-inflammatory, immunomodulatory, or antioxidant could help to reduce neurodegenerative processes [38,45].

Therefore, it is essential to consider that although this species has impressive potential for psychiatric and neurological disorders, much remains to be done.

## 12. Conclusions

*Coriandrum sativum* is a widely edible and medicinal plant identified as GRAS. This species has been studied as a therapeutic resource for experimentally treating psychiatric and neurological disorders due to its relevant activities in the central nervous system. The information collected in this review shows that terpenes and flavonoids, compounds mainly identified in the plant, exhibit different mechanisms of action on the receptors of the neurotransmitters GABA, glutamate, and serotonin, involved in the development of psychiatric disorders. These bioactive molecules offer a competitive advantage in the research and development of a phytomedicine using a standardized extract of *C. sativum*. The main strength is the broad therapeutic approach, as it comprises components directed to multiple pharmacological targets and with different modes of action. However, it is necessary to investigate the complex interaction between the several active components with the different pharmacological mechanisms through methodologies like molecular docking, in vitro linkages, in vivo behavioral studies, and their pharmacological characteristics discussed here. The research provided in this review will help in the establishment of a *C. sativum*-based clinical treatment helpful to counteract the pathophysiological picture that underlies neurological diseases, such as epilepsy, or psychiatric disorders, like anxiety and depression, with a great impact on the well-being of society.

## Figures and Tables

**Table 3:** Summary of molecular docking studies with *C. sativum* compounds.

Pathology	Pharmacological Model	Biological Targets	*C. sativum* Compounds	Results	Reference
Leukemia	Chronic (K562) and acute (HL60) myeloid leukemia cell lines and on normal Vero cell line	ABL kinase ABL1 Bcl2 FLT3	Epicatechin gallate, epicatechin, catechin rutin, vanillic acid	Activity on receptor: ABL kinase: similar effect for epicatechin and rutin ABL1: epicatechin gallate > rutin Bcl2: epicatechin gallate > vanillic acid FLT3: catechin > vanillic acid	[147]
Diabetic neuropathy	Diabetic neuropathy induced by streptozotocin	TNF-α	Quercetin, kaempferol, rutin	Quercetin> rutin> kaempferol	[148]
Diabetic nephropathy	Diabetic nephropathy in streptozotocin nicotinamide induced type 2 diabetes model	Receptors of AGEs	Linalool	Suitable binding interaction with amino acid residues of RAGEs (Dock score = −2.098 kcal/mol)	[149]
Diabetic nephropathy	Diabetic nephropathy induced by streptozotocin	KCNQ2/KCNQ3	(E)-2-dodecenal	Binding in a juxtaposed zone between residues on the KCNQ S5 transmembrane segment and S4-5 linker	[89]
Hyperglycemia	In vitro methods for aldose reductase (ALR) enzyme inhibition, antiglycation activity, and sorbitol accumulation inhibition	ALR, receptors of AGEs, and sorbitol dehydrogenase	Flavonoid-rich seeds extract: Quercetin, rutin, kaempferol, (+)-catechin, luteolin, rhamnetin, and apigenin	Rutin, (+)-catechin, and rhamnetin exerted a suitable binding with all receptors with a docking score up to −4.94 for sorbitol dehydrogenase, and mostly hydrogen bonding and hydrophobic interactions were found with ALR enzyme receptors	[150]

## Data Availability

The data presented in this review are available from the search databases described in the methodology.
